# The Trained Sniffer Dog Could Accurately Detect the Urine Samples from the Patients with Cervical Cancer, and Even Cervical Intraepithelial Neoplasia Grade 3: A Pilot Study

**DOI:** 10.3390/cancers12113291

**Published:** 2020-11-06

**Authors:** Akihito Yamamoto, Seiryu Kamoi, Keisuke Kurose, Marie Ito, Toshiyuki Takeshita, Shoko Kure, Katsuichi Sakamoto, Yuji Sato, Masao Miyashita

**Affiliations:** 1 Department of Obstetrics and Gynecology, Nippon Medical School, Tokyo 113-8603, Japan; skamoi@nms.ac.jp (S.K.); kuro@nms.ac.jp (K.K.); yasuimarie@nms.ac.jp (M.I.); toshimac@nms.ac.jp (T.T.); 2 Department of Pathology and Integrative Oncological Pathology, Nippon Medical School, Tokyo 113-8602, Japan; skure@nms.ac.jp; 3 Department of Gynecology, Jizankai Medical Foundation Tsuboi Cancer Center Hospital, Fukushima 963-0197, Japan; ksakamo@xc4.so-net.ne.jp; 4 Cancer Sniffing Dog Training Center, St. Sugar Japan, Chiba 294-0226, Japan; power_of_dog_satoh@yahoo.co.jp; 5 Nippon Medical School, Tokyo 113-8602, Japan; miyashit@nms.ac.jp; 6 Twin Peaks Laboratory of Medicine (TPLM), Twin peaks Laboratory of Medicine, Yamagata 999-4331, Japan

**Keywords:** cervix uteri, dogs, odorants, urine, volatile organic compounds

## Abstract

**Simple Summary:**

Tumor detection by dog sniffing is a possible new method in cancer diagnosis. The aim of this study is to assess whether a trained dog can distinguish urine samples from cervical cancer patients. Urine samples were collected from 34 with cervical cancer, 49 patients with CIN3, 49 with benign uterine diseases, and 63 healthy volunteers. In all 83 test runs, one positive sample among five samples were presented to the dog. The trained dog accurately distinguished the urine sample of a cancer patient from those of the controls. This study showed that cancer detection by dog sniffing can be a non-invasive, cost-effective screening technique for cervical cancer.

**Abstract:**

(1) Background: Previous reports have indicated that cancers of the stomach, lung, and pancreas can be detected by dog sniffing, but results have been varied. Here, a highly trained dog was used to determine whether urine from patients with cervical premalignant lesions and malignant tumors have a cancer-specific scent. (2) Methods: A total of 195 urine samples were collected from patients with cervical cancer, cervical intraepithelial neoplasia grade 3 (CIN3), benign uterine diseases, and healthy volunteers. Each test was performed using one urine sample from a cancer patient and four samples from different controls. Each of the five urine samples was placed in a separate box. When the cancer sniffing dog stopped and sat in front of the box with a sample from a cancer patient, the test was considered as positive. (3) Results: 83 patients with cervical cancer (34 cases of cervical cancer and 49 cases of cervical intraepithelial neoplasia grade 3), 49 patients with uterine benign diseases, and 63 healthy volunteers were enrolled, and their urine samples were collected. In 83 times out of 83 runs in a double-blind test, the trained dog could correctly identify urine samples of cervical cancer patients. (4) Conclusion: A trained dog could accurately distinguish the urine of all patients with cervical cancer or CIN3, regardless of the degree of cancer progression.

## 1. Introduction

In spite of continued progress in diagnosis and treatment of gynecological cancers, they still remain one of the top causes of cancer-related morbidity and mortality globally. Although cervical cancer screening using conventional and/or liquid-based cytology is recommended for population-based and opportunistic screening with sufficient evidence [1,2], the screening rate among Japanese women is approximately 20%, which is much lower than for women in western countries, possibly due to privacy issues or pain associated with the procedure [3,4]. Screening using either human papillomavirus testing or a serum tumor marker is not recommended as a population-based screening [5]. Given the drawbacks of the current screening methods, an alternative, non-invasive screening technique with high accuracy and low cost is needed.

Cancer detection by dog sniffing is a possible new method to detect cancer. The first report on the use of a house dog to detect melanomas was very novel and suggested that a distinct scent was released from tumors [6]. Subsequent reports have described dogs detecting various types of cancer in both urine and breath samples by sniffing [7,8,9,10]. Canine cancer detection of cervical cancer was first reported in 2017 [11]. In the study, a trained sniffer dog was able to detect cervical cancer using the fresh cervical smear samples. Attempts to detect cervical cancer from a urine sample by dog sniffing has not been reported.

We hypothesized that scent trained dogs could distinguish between cervical cancer patients and non-cervical cancer patients using urine samples. The aim of this study was to investigate the efficacy of a cancer detection method by sniffing dogs using urine samples of patients diagnosed with cervical cancer.

## 2. Results

### 2.1. Clinical Characteristics of the Patients

A total of 195 patients were enrolled in this study, including 83 cases of cervical cancer patients (49 cases of cervical intraepithelial neoplasia, grade 3 and 34 cases of cervical cancer), 49 cases of benign uterine diseases (uterine leiomyoma, 39 cases; uterine endometriosis, 8 cases; and uterine prolapse, 2 cases), and 63 healthy controls. The number and ages of the patients are shown in Table 1. Age distribution did not vary among the three groups (*p* = 0.915).

Clinical stages of the cervical cancer patients were Stage I (10 cases), II (4 cases), III (14 cases), and IV (6 cases). Pathological diagnoses were squamous cell carcinoma (27 cases), adenocarcinoma (five cases), and undifferentiated tumor (2 cases) (Table 2).

### 2.2. Condition of the Dog During the Detection Test

A total of 83 runs were carried out for the detection test. Throughout the entire testing period, no adverse events, injury or illness were experienced by the dog. The concentration level of the dog was high in all runs.

### 2.3. Sensitivity and Specificity of the Detection Test

Urine samples from all cervical cancer or CIN3 patients were positive by the dog sniff test. Urine samples from all benign uterine diseases or healthy persons were negative (Table 3).

Among patients with cervical cancer and the control group, sensitivity and specificity of canine scent detection of urine samples compared with conventional histopathological diagnosis were both 1.00.

## 3. Discussion

Feasibility of a trained dog to distinguish between urine samples of cervical cancer and CIN3 from urine samples of a control group comprised of benign uterine diseases and healthy volunteers could be demonstrated. Using our established method, the dog could detect urine samples of cervical cancer and CIN3 with 100% sensitivity and specificity in the double-blind test series. This is the first study to show the high accuracy of cervical cancer detection by a trained dog using urine samples.

Accuracy of canine scent detection as reported in published articles varies. Willis et al. reported that dogs can distinguish urine from bladder cancer patients with a mean success rate of 41% [8], while McCulloch et al. reported that ordinary household dogs can be trained to distinguish breath samples of patients with lung and breast cancer from those of control volunteers with a sensitivity and specificity of 0.99 and 0.99 for lung cancer, and 0.88 and 0.98 for breast cancer [9]. Moreover, Horvath et al. compared ovarian cancer tissue with control tissue and reported a sensitivity of 100% and specificity of 97.5% [10]. In the series of these studies, inter-dog difference may exist. In this study, an extremely high specificity was shown using urine samples from cancer patients and controls. Since the sense of smell of the test dog is critical, the training method used in the previous trial [12], which is well-established, was applied. Furthermore, in order to maintain the performance of the dog, the test environment, such as temperature and humidity, was strictly monitored in the current test-runs.

Originally, dog training for cancer detection was carried out using the breath of a cancer patient. We could subsequently apply it to urine samples in the pre-tests. Next, we hypothesized that the trained dogs could distinguish between cervical cancer patients and non-cervical cancer patients using urine samples. Collection of a urine sample is non-invasive and cost-effective. In addition, the ease of collection and handling renders urine samples ideal for future screening of cervical cancer. Cornu et al. reported prostate cancer detection using urine samples by dog sniffing [13]. Urine samples from 33 prostate cancer patients and 33 healthy controls were tested, and the trained dogs could detect prostate cancers by sniffing urine with sensitivity and specificity of 91%. Up to now, no reports on cervical cancer detection using urine samples by dog sniffing have been published. The results of this study demonstrate the feasibility of a canine cancer detection method for cervical cancer using urine samples with high sensitivity and specificity for the first time. The potential of canine scent detection is considered to be a practical and accurate method for routine detection of cervical cancer. The results of this study indicate the presence of volatile biomarkers for cervical cancer and CIN3, and identifying these biomarkers may be an ideal method for non-invasive, early detection of cervical cancer.

Volatile organic compounds (VOCs) are emitted throughout the body, including breath, blood, and urine. Production of VOCs by the human body differs greatly by individual, since VOCs are influenced by genetic background, environmental factors, and medical history, such as a comorbidity [14]. However, VOCs have been associated with many cancer types [15,16,17,18,19,20,21,22,23,24,25,26,27], and it has been suggested that they can be used as alternative cancer screening markers. Canine cancer detection, as well as the recent Caenorhabditis elegans cancer detection method, are believed to be based on these cancer-related VOCs [18,28]. Still, clarification of the exact chemical compounds and/or their combinations in cervical cancer is needed. Further study in order to develop of a novel diagnostic method for cancer based on VOCs is desired.

Because CIN3, carcinoma in situ as well as pre-cancerous lesions, could be detected in this study, canine cancer detection is useful for early identification of cancer development and intervention using preventative or therapeutic agents. Such interventions could markedly reduce the cost of treating cancer and have life-saving benefits. The fact that all CIN3 cases in this study had a positive response regardless of the lack of tumor cell invasion, supports the idea that specific VOCs are emitted from the early stages of malignant cell growth. Tumor cells, immune cells, and other cell types are thought to release VOCs [27], and thus both rapidly growing tumor cells as well as inflammatory cells may release specific VOCs which could be detected by the dog. Another report suggested that VOCs may be metabolic products of oxidative stress [29,30]. However, the mechanism on how cancer-specific VOCs are produced has yet to be elucidated.

Persistent infection of the cervix with certain high-risk types of human papillomavirus (HPV) has been well established as a cause of cervical cancer [31]. High-risk types of HPV, especially types 16 or 18, are identified in nearly all cervical cancers [32,33]. The interaction between HPV infection and VOC production has not yet been evaluated. Screening for HPV infection by detecting HPV DNA in urine samples was first investigated in 1991 [34]. Considering the different stages of infection and possible cell disintegration, HPV DNA can appear in urine as being integrated in the cellular genome (cell-associated or cell-free), in the form of intracellular episomal DNA, cell-associated viral DNA-containing particles, free viral DNA-containing particles, or free viral DNA. It will be of great interest to investigate HPV DNA in positive urine samples collected in this study to provide evidence for the interaction between HPV infection and VOC production.

This study has several limitations. First, in the current study, urine samples from CIN1 and CIN2 patients were not included. Currently, in daily practice, CIN1 and CIN2 lesions need follow-up. For future study, detection of CIN1 and CIN2, and distinguishing these categories are helpful. Second, this study used a single dog. The dog used in the study was the most capable dog at the training center. She was initially trained as a salvage dog, but her ability was found to be far superior to other dogs. Not all dogs become proficient at sniffing out cancer odors, and as a result, it may be difficult to recruit dogs with excellent ability. Third, training the dogs incurs costs and requires time at an appropriate facility. It takes at least a year to train a dog. For effective dog training with the established method, cooperation with dog-training centers and expert trainers is needed.

In the clinical practice, there might be unknown numbers of positive and negative samples for the screening. Additionally, in the current study setting, there is a concern that the dog is recognizing the unique sample rather than detecting cancer. Therefore, the next study should contain test-runs of different numbers of positives and controls, including all positives and all controls, with a larger number of controls so that they are used only once. Unfortunately, this trained dog died, making it difficult to perform a follow-up test immediately. We are now training other dogs and are preparing for additional experiments for further study.

We hope the results of our present study, as a pilot study, provides the development of a new cancer screening method of cancer detection. Our next step is to conduct additional tests with enlarged case numbers with several other trained dogs for reproducibility and prevalence. However, for worldwide use, our feature goal is to detect cancer-specific volatile organic compounds, which the dog sensed, using gas chromatography for the new screening method.

## 4. Materials and Methods

### 4.1. Cancer Patients and Control Sample Donors

Patients diagnosed with either cervical cancer or CIN3 and healthy volunteers at Nippon Medical School Hospital or the Jizankai Medical Foundation Tsuboi Cancer Center Hospital between January 2011 and October 2012 were enrolled. In all cancer cases and benign disease cases, a definitive diagnosis was obtained by histopathological evaluation of a biopsy or resected tissue. Patients who received a surgical operation before urine sample collection, and those with other types of cancer were excluded. Healthy volunteers were verified with systematic cancer screening tests including blood test, chest X-ray, abdominal ultrasound, mammography, and gynecological examination. This study was conducted in accordance with the principles embodied in the Declaration of Helsinki, and its protocol was approved by the Ethics Committees of Nippon Medical School Hospital (Tokyo, Japan) and the Jizankai Medical Foundation Tsuboi Cancer Center Hospital (Fukushima, Japan) (IRB#23-03-156). A written explanation of the study was provided to all potential participants and each was enrolled after providing written consent.

### 4.2. Urine Sample

For patients with either cervical cancer or CIN3 lesions, spontaneously voided urine was collected into a paper cup (Harn cup laminate A; Nissho Sangyo Co., Ltd., Tokyo, Japan) one day prior to surgery or concurrent chemoradiotherapy, and then transferred into sterile test tubes (Sterile SP tube, Eiken Chemical Co., Tokyo, Japan). Each test tube was sealed with a cap and then stored at −80 °C until 1 mL of the selected sample was used for the dog sniffing test. Spontaneously voided urine from the control participants was collected and stored in the same manner. Urine samples were used in the test for up to 6 months after collection. Frozen urine samples were thawed and used for the test at room temperature.

### 4.3. Dog and Training

**Figure 1:**
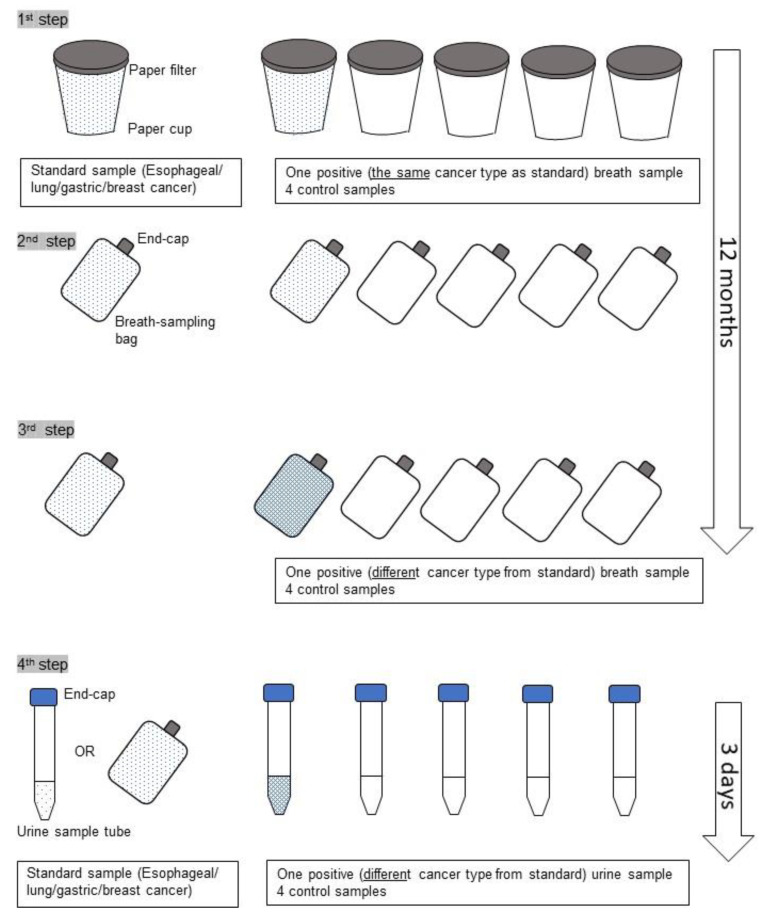
The four steps of the training are shown. In the first and the second step, the dog tried to detect the breath sample, which is the same cancer type as the standard. In the third step, the positive breath sample was a different cancer type from the standard. In the fourth step, urine samples were used.

A single dog, named Marine, kept in the kennel at the Cancer Sniffing Dog Training Center, St. Sugar Japan, in Tateyama City, Chiba, Japan was used in this study. This study was carried out in strict accordance with the recommendations in the Guide for the Care and Use of Laboratory Animals of the United States National Institutes of Health. The protocol was approved by the Institutional Ethics Committee of Nippon Medical School Hospital (IRB#23-03-156). Dog selection in this study was critical. The dog had passed preliminary tests confirming the ability to selectively sniff both the breath and urine of cancer patients [12]. Originally, the dog was a water rescue dog, and then, she was trained by a professional trainer for cancer detection. The training method is described in the previous study, in which the same dog was used [12]. Briefly, the dog was a 9-year-old female Labrador retriever recruited from the Cancer Sniffing Dog Training Center [12]. Initially, she was trained to sniff the breath of patients with different types of cancers. The breath samples and urine samples used in the training steps were collected from several hundred cancer patients and about 500 healthy volunteers recruited using the internet. The training consisted of four steps (Figure 1). In the first step, breath samples from one patient with esophageal cancer and four controls were used. The samples were placed into a paper cup from the breath sampling bags with a paper filter. After 2 days, the five sample cups were placed on the floor. First, the trainer let the dog smell the standard breath sample from the patient with esophageal cancer, and then let her identify the cup containing the esophageal cancer breath from the five sample cups. If she correctly identified the cancer sample, she was rewarded with a tennis ball. A similar training session was conducted using breath samples from patients with lung cancer and gastric cancer on the following days. In the second step, the breath-sampling bag was used with the end cap on. Breath-sampling bags contained esophageal, lung, or gastric cancer scents. In the third step, the dog first smelled the standard breath sample, and then the trainer let her identify the bag with other cancer types. The training was then continued by adding samples of other types of cancers as well as controls. It took about 12 months for these steps. In the fourth step, the dog was trained with the urine samples. First, she smelled the standard urine sample of either esophagus, lung, gastric cancer, or breast cancers. Then, she tried to detect urine samples from other cancer types. It took only three days to accomplish the training. During the training, she was able to use breath sample as a standard. In this way, she was already able to detect urine samples of the following cancer types: esophageal cancer, breast cancer, lung cancer, gastric cancer, pancreatic cancer, hepatocellular carcinoma, cholangiocarcinoma, colorectal cancer, prostate cancer, uterine cancer, ovarian cancer, and bladder cancer, before the present study. When she could not stay focused, the dog sniffing tests would not be conducted. Conditions on days when the test could not be conducted included days with extremely hot and humid weather.

### 4.4. Test Boxes

**Figure 2:**
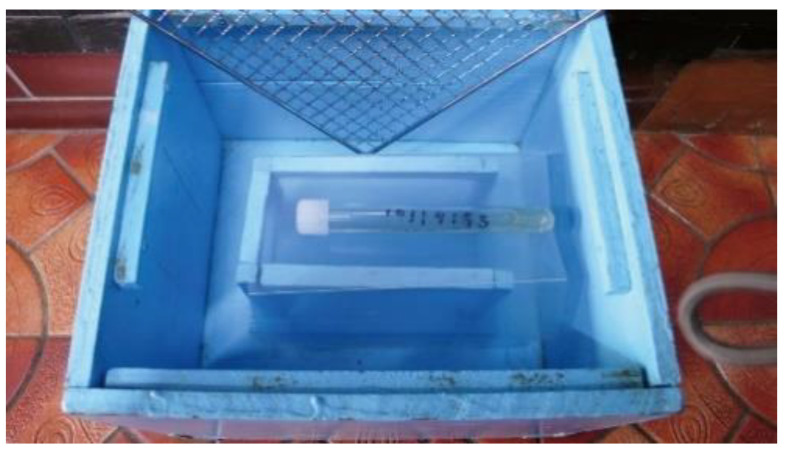
Test Box. The test tube sample with the end cap on was placed in this box.

The test boxes were wooden storage containers painted light blue and measuring 27 cm wide, 30 cm deep, and 20 cm long (Figure 2). Inside each box was fitted with a 10 cm deep wall on which a urine sample tube could be placed. So the dog would not come in direct contact with the test sample, each box was covered by a metal mesh. When tests were conducted, the five test boxes were placed in a straight line on the floor at a distance of 1 m apart.

### 4.5. Detection of Urine Samples from Cervical Cancer and CIN3 Patients

**Figure 3:**
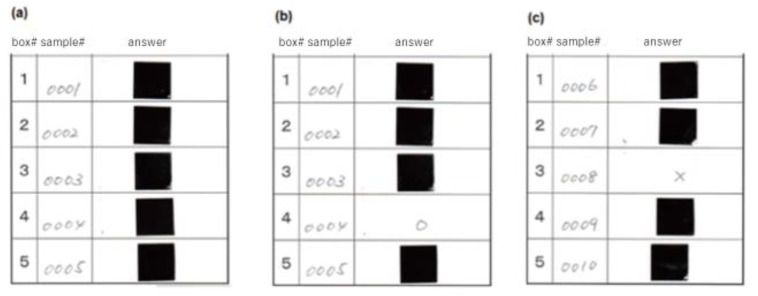
Answer Sheets used in the test. The test sample numbers and box numbers were shown on the answer sheet (**a**). The box number for the cancer sample was identified by an adjacent circle (**b**) and box numbers for the other samples were marked with an adjacent cross (**c**). The marks were then covered by a non-reattachable sticker. Examples of right (**b**) and wrong (**c**) answers are shown.

Procedures to detect urine samples from cervical cancer and CIN3 patients were similar to those used to detect breath samples from cancer patients. In brief, test tubes containing new urine samples from cervical cancer and CIN3 patients, as well as those from healthy controls, were used in each test. The tubes were kept separate to avoid any possibility of contamination of the control samples with potentially volatile organic compounds (VOCs) from the cancer and CIN3 samples. A chart to randomize numbers was used to determine the order in which the urine samples were placed in the boxes. The number was written on the sample and converted from a serial number to a test number by a third party at the same time. The test number and test box number were recorded on an answer sheet. Since the dog was to be rewarded for a correct response by playing with the tennis ball, the answer had to be known as quickly as possible by the trainer and the dog, so an answer card format was used. On the answer sheet, the urine sample from patients with cervical cancer or CIN3 was identified by an adjacent circle next to the test box number, and urine samples from the control patients were marked with an adjacent cross. The marks were then covered with a sticker, which once detached, could not be reattached (Figure 3).

**Figure 4:**
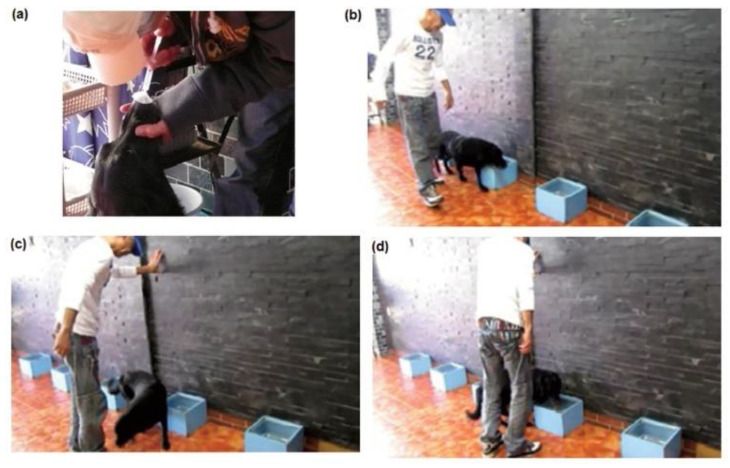
Cancer Detection of Urine Samples. The dog sniffs a 5-mL breath sample from a cancer patient that had been prepared beforehand (**a**), then walked past each test box (**b**), turned around (**c**), and then sat in front of the box which was considered to contain the cancer sample (**d**).

The assistant placed the test tube samples in the boxes according to the number noted on the answer sheet. The sample content was unknown to the dog, the trainer, and the assistant; thus, the study was double-blinded. At the beginning of the test, after the dog had been trained to concentrate, the dog’s nose was exposed to a 5-cc breath sample from breast cancer patient used in the training steps described above. The trainer then attached a leash and walked the dog by the test boxes to permit her to sniff the urine samples. The dog was expected to sit in front of the box containing urine from either a cervical cancer or CIN3patient (Figure 4).

The assistant peeled off the sticker next to the box number on the answer sheet and checked the results. The answer sheets were collected by mail and checked to verify whether the test had been conducted correctly. For each test, the dog’s concentration level (high, normal, or low) was assessed and recorded. Tests were always held on days when the dog’s concentration level was high. Tests were not conducted during extreme environmental conditions such as days with high temperature and humidity, or during irregular natural phenomena such as earthquakes and typhoons, as the dog’s concentration level at such times was low.

### 4.6. Evaluation of the Dog’s Response

The dog sat in front of the positive samples after sufficiently smelling all five boxes in each test run. The verdict of the test was determined after confirming that the dog did not move spontaneously for three seconds. If the dog started to move before that time, the test verdict was temporarily suspended. In such case, assessment was determined when the dog sat in front of the test box and did not move for three full seconds. Dog responses were categorized into two correct and two incorrect actions. Correct actions were (1) sitting down in front of a sample box containing a urine sample from a patient with cervical cancer or CIN3 (true positive in sensitivity calculations) and (2) only sniffing a control sample and not sitting in front of it (true negative). Incorrect actions included (1) sitting in front of a control sample (false positive) and (2) not sitting in front of a sample from a patient with cervical cancer or CIN3 (false negative).

### 4.7. Staging and Statistical Analysis

Evaluation of the stage of cervical cancer was carried out based on criteria described by the International Federation of Gynecology and Obstetrics. Diagnostic accuracy was calculated as the sensitivity (or the true positive rate) and specificity (or the true negative rate) of the dog’s identification of positive urine samples as compared to diagnosis by conventional histopathological methods for either cervical cancer or CIN3 lesions. Results were expressed as the mean ± standard deviation. Normality of distribution was examined for age, which was a continuous data. Analysis of variance was used to compare age between the three groups, where the data showed normal distribution. The test was two-sided, and *p* < 0.05 was considered statistically significant. Statistical analysis was performed with EZR (Saitama Medical Center, Jichi Medical University, Saitama, Japan) [35].

## 5. Conclusions

In conclusion, this study demonstrated that a trained dog can successfully distinguish urine samples of cervical cancer and CIN3 patients from those of benign uterine diseases and healthy volunteers. Canine cancer detection can be a non-invasive, cost-effective screening technique for cervical malignancies in women.

## Figures and Tables

**Table 1:** Patients by group and age.

Diagnosis (numbers)	Cx. Cancer *	Benign	Healthy	*p* Value
	*n* = 83	*n* = 49	*n* = 63	
Age (years)	44 ± 15	44 ± 8	44 ± 10	0.915
Mean ± SD				

Cx. Cancer, cervical cancer; Benign, benign uterine disease; Healthy, healthy control. * cervical cancer included cervical intraepithelial neoplasia grade 3.

**Table 2:** Clinical stage and pathological diagnosis of cervical lesions/tumors.

Clinical Stage	Numbers
CIN3	*n* = 49
Stage I	*n* = 10
Stage II	*n* = 4
Stage III	*n* = 14
Stage IV	*n* = 6
Pathological diagnosis	
CIN3	*n* = 49
SCC	*n* = 27
Adeno	*n* = 5
Others	*n* = 2

CIN3, cervical intraepithelial neoplasia grade 3; SCC, invasive squamous cell carcinoma; Adeno, adenocarcinoma; Others, undifferentiated carcinoma.

**Table 3:** Results from canine scent detection of urine samples from different patient groups.

Diagnosis	Result	Numbers
CIN3 (*n* = 49)	+	49
	−	0
Cervical Cancer (*n* = 34)		
Stage I (*n* = 10)	+	10
	−	0
Stage II (*n* = 4)	+	4
	−	0
Stage III (*n* = 14)	+	14
	−	0
Stage IV (*n* = 6)	+	6
	−	0
Benign Uterine Disease		
Leiomyoma (*n* = 39)	+	0
	−	39
Endometriosis (*n* = 8)	+	0
	−	8
Uterine prolapse (*n* = 2)	+	0
	−	2
Healthy control (*n* = 63)	+	0
	−	63

CIN3: cervical intraepithelial neoplasia grade 3.
